# Oropouche virus: Understanding “sloth fever” disease dynamics and novel intervention strategies against this emerging neglected tropical disease

**DOI:** 10.1080/21505594.2024.2439521

**Published:** 2024-12-13

**Authors:** Michaela Cain, Hinh Ly

**Affiliations:** Department of Veterinary & Biomedical Sciences, College of Veterinary Medicine, University of Minnesota, Twin Cities, MN, USA

**Keywords:** Oropouche virus, OROV, arbovirus, bunyavirus, sloth fever, acute febrile illness

## Abstract

Oropouche virus (OROV), an arbovirus belonging to the Orthobunyavirus genus and *Peribunyaviridae* family, is the causative agent of the so-called “sloth fever.” The virus primarily relies on the midge vector *Culicoides paraensis* for transmission, maintaining both sylvatic and urban cycles. Human infections are characterized by acute febrile symptoms, and severe cases can lead to neurological complications. Since its first isolation in 1955, OROV has caused numerous outbreaks throughout South America, infecting over half a million people. Recent outbreaks in the Amazon and the Caribbean, along with cases reported in U.S. travellers, underscore the growing threat of OROV amid climate change and increased global travel. With no FDA-approved vaccines or specific antiviral treatments available, current management of the disease caused by OROV infection is limited to supportive care. The urgent need for effective vaccines is amplified by the potential for geographic expansion of the virus and its transmitting vector(s). The ongoing development of OROV vaccine candidates represents a crucial step towards controlling future OROV outbreaks and enhancing global public-health preparedness against this emerging infectious disease.

The OROV genome consists of three negative, single-stranded RNA segments: the Large (L) segment, which encodes the RNA-dependent RNA polymerase (RdRp); the Medium (M) segment, which encodes the surface glycoproteins (Gn and Gc) and the non-structural protein NSm; and the Small (S) segment, which encodes the nucleocapsid protein (N) and the non-structural protein NSs [1,2]. Due to its trisegmented genomic organization, OROV is susceptible to reassortment, which is an important driver of genetic divergence and diversity [3].

OROV is an arbovirus that primarily relies on the midge vector *Culicoides paraensis* for transmission, with some mosquito vectors, including *Culex quinquefasciatus*, *Aedes aegypti*, and *Ochlerotatus serratus*, also contributing to its spread. OROV is maintained in two transmission cycles: a sylvatic cycle and an urban cycle (Figure 1a). The sylvatic cycle is thought to involve reservoir hosts such as three-toed sloths (and hence the term “sloth fever” as a disease caused by this virus), New World non-human primates (e.g. capuchin and howler monkeys), and wild birds, although the primary reservoir host has not been definitively identified [1]. The urban cycle, responsible for sudden OROV outbreaks, is primarily driven by bites of the infected adult female midges. To date, there have been no reported cases of human-to-human transmission of Oropouche (“sloth”) fever^2^.

**Figure 1.**
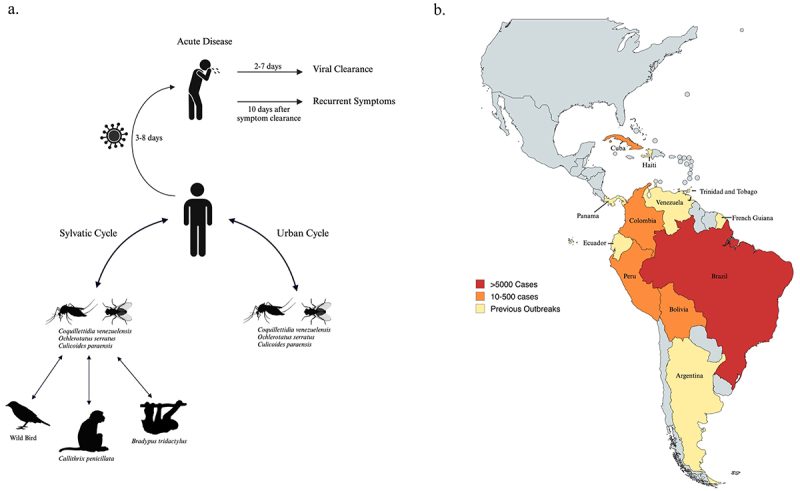
Transmission dynamics and geographic distribution of oropouche virus (a) transmission cycle of oropouche virus (OROV). The transmission of OROV occurs through sylvatic and urban cycles. After a bite from an infected insect, the incubation period of OROV is 3–8 days. Symptoms generally last for 2–7 days, but in cases of persistent infection, they can extend to 2–4 weeks. If disease recurrence occurs, it typically happens within 10 days of the initial clearance of symptoms. (b) Map of oropouche virus (OROV) outbreaks in 2024 and historic outbreaks in endemic countries in South America. Data reported by the pan American health organization show the number of OROV cases per country as of July 2024. Regions with previous OROV outbreaks without known current cases are highlighted in yellow.

OROV was first isolated in a forest worker in Trinidad in 1955 [4]. Five years later, OROV was isolated from mosquitoes in Trinidad, confirming them as an important reservoir [4]. The first recoded OROV outbreak occurred in Brazil in Belém, Pará in 1961, infecting approximately 11,000 people [4]. Since then, there have been more than 30 “sloth fever” epidemics reported in Pará and throughout the Brazilian Amazon region (Figure 1b) [5]. It is estimated that over half a million people throughout South America have been infected with OROV since its initial isolation, though this number is likely underestimated due to the clinical similarity of OROV symptoms to other arboviral febrile illnesses, such as Dengue, Zika, and Chikungunya [2,4].

Following the bite of an infected midge or mosquito, the incubation period for OROV ranges from 3 to 8 days before symptoms begin to manifest [1]. Common symptoms include headache, retro-orbital pain, malaise, myalgia, arthralgia, nausea, vomiting, and photophobia. In severe cases, OROV infection can lead to meningitis, encephalitis, dizziness, anorexia, and other systemic complications [3]. For most affected individuals, the acute phase of the disease is relatively short, lasting between 2 and 7 days, although symptoms can persist for 2 to 4 weeks in some cases. Up to 70% of patients have reported the reoccurrence of symptoms, which typically occurs within 10 days of initial symptom clearance [6]. The host’s innate immune response, particularly the production of interferons and pro-inflammatory cytokines, is critical in controlling and resolving OROV infection [1].

In early 2023, large outbreaks of OROV infection were reported in the Amazon region, with new outbreak areas also identified in South America and the Caribbean (Figure 1b) [6,7]. In July 2024, the first case of OROV transmission from a mother to foetus (vertical transmission) was documented and linked to adverse pregnancy outcomes, including foetal death and congenital abnormalities, such as microcephaly, a rare neurological condition in which an infant’s head is deformed and significantly smaller than the head of other children of the same age [6]. According to the Centers for Disease Control and Prevention (CDC), as of 16 August 2024, 21 cases of OROV infection have been confirmed in U.S. travellers returning from Cuba [6]. In response to the increased OROV activity, the CDC issued a Health Advisory warning of the rising risk to travellers [6].

The increased accessibility of global travel, along with increased deforestation and urbanization of endemic regions, and the expanding geographic range of the virus-transmitting vectors due to climate change, underscore the need to prioritize research on this neglected tropical disease [8]. There are no FDA-approved vaccines or specific antiviral treatments for OROV, and clinical management is limited to supportive care aimed at alleviating symptoms. There are also no clinically validated diagnostic tests for OROV, and the virus is not routinely screened for in endemic regions, leading to underdiagnosing and underreporting of cases [6]. This lack of therapeutic options and diagnostic tools highlights a critical gap in addressing OROV infections. Given the virus’s potential to cause outbreaks in new regions, the development of effective clinical and diagnostic tools is essential.

There is one vaccine candidate against OROV currently in development: a recombinant vesicular stomatitis virus (rVSV) vector expressing the OROV glycoproteins Gn and Gc [9]. A recent study identified several potential vaccine epitopes within the M-segment polyprotein that can stimulate strong immune responses against the virus [10]. A reverse genetics system for OROV, developed in 2016, provides researchers with a powerful tool to manipulate the viral genome, allowing for a better level of understanding of the mechanisms of viral replication, pathogenesis, and immune response. This system also enables the generation of attenuated or modified viruses that could be used as vaccine candidates or in studying immune responses to the infection [11]. Several challenges hinder the development of an OROV vaccine, including the high genetic variability between virus strains, lack of primary research on viral pathogenesis in both the vector host(s) and humans, and inadequate disease modelling in comparative animal models [1,8]. Additionally, because OROV infections rarely cause lethal disease, the virus often gets overlooked. However, despite its generally non-lethal nature, OROV infection can cause recurring symptoms that significantly contribute to the disease burden in developing nations. In addition, the spread of insect vectors to new regions, and the ongoing outbreaks in the Americas further highlight the need for preventative research.

Addressing the burden of non-lethal neglected tropical diseases like “sloth fever” that is caused by OROV is crucial not only for mitigating future outbreaks and enhancing global public health preparedness but also supports socio-economic development in affected regions. Reducing the prevalence of such disease can improve quality of life, allowing individuals to contribute more effectively to their families, communities, and economies. Integrating efforts to combat these neglected tropical diseases within the One Health framework, which considers the interconnectedness of human, animal and environmental health, will be essential for creating a comprehensive and sustainable approach to disease prevention and control globally.

## Data Availability

No primary data are included in this article.
